# Tomato Leaf Disease Detection Method Based on Multi-Scale Feature Fusion

**DOI:** 10.3390/plants14203174

**Published:** 2025-10-16

**Authors:** Xiangrui Meng, Cong Chen, Wenxue Dong, Ke Wang

**Affiliations:** 1 School of Big Data and Artificial Intelligence, Chengdu Technological University, Chengdu 611730, China; mxrui1@cdtu.edu.cn (X.M.);; 2 College of Information and Communication Engineering, Hainan University, Haikou 570228, China; cclengdong@163.com; 3 College of Mechanical and Electrical Engineering, Inner Mongolia Agricultural University, 306 Zhaowuda Road, Saihan District, Hohhot 010018, China

**Keywords:** tomato leaf disease, YOLO11n, EfficientMSF module, C2CU module, CAFMFusion module

## Abstract

Tomato is a key economic crop whose yield and quality depend heavily on the early and accurate detection of leaf diseases. Conventional diagnosis based on manual observation is labor-intensive and prone to subjective bias. To overcome the limitations of disease detection under complex environmental conditions, this study presents an enhanced YOLO11n-based detection framework for tomato leaf diseases. The proposed model integrates an EfficientMSF module in the backbone to strengthen multi-scale feature extraction, introduces a C2CU module to enhance global contextual representation, and employs a CAFMFusion module to achieve efficient fusion of local and global features. Experiments were conducted on a self-constructed dataset containing nine tomato leaf categories, including eight disease types and healthy samples. The proposed approach achieves an average Recall of 71.0%, mAP@0.5 of 76.5%, and mAP@0.5–0.95 of 60.5%, outperforming the baseline YOLO11n by 3.4%, 1.3%, and 2.0%, respectively. In particular, for the challenging Leaf Mold class, mAP@0.5 improved by 3.4%. These results demonstrate that the proposed method possesses strong robustness and practical applicability in complex field conditions, offering an effective solution for intelligent tomato disease monitoring and precision agricultural management.

## 1. Introduction

Tomato (*Solanum lycopersicum*), one of the most widely cultivated economic crops worldwide, is grown extensively in both greenhouse and open-field environments. Its yield and quality play a crucial role in determining agricultural productivity and economic returns. However, tomatoes are highly susceptible to various foliar diseases, including early blight, late blight, and leaf mold, which can cause leaf discoloration, wilting, and even complete plant death, ultimately resulting in substantial reductions in yield and quality [1,2]. Consequently, the early detection and precise management of tomato diseases are essential for ensuring sustainable crop production and minimizing economic losses [3].

Traditional approaches to plant disease diagnosis primarily rely on manual observation by agricultural experts or farmers. However, the scalability and efficiency of such methods are severely limited by geographic and operational constraints. In large-scale farming systems or across extensive agricultural regions, comprehensive field inspections are prohibitively time-consuming, labor-intensive, and economically unsustainable, making continuous monitoring impractical. Even in localized studies, the scarcity and uneven distribution of plant pathology experts hinder consistent diagnosis. Moreover, diagnostic accuracy often varies among individuals and is influenced by subjective judgment and environmental conditions, leading to inconsistent results and potential misdiagnoses. In recent years, advances in computer vision and artificial intelligence have catalyzed a paradigm shift toward automated plant disease detection [4,5]. Deep learning–based methods, particularly those employing Convolutional Neural Networks (CNNs), have demonstrated remarkable success in image classification and object detection, providing an efficient and objective alternative to manual assessment [4,6]. Among these, the You Only Look Once (YOLO) family of detectors has gained prominence for agricultural applications due to its end-to-end architecture, real-time detection capability, and high accuracy [7,8,9,10,11,12,13]. YOLO can simultaneously localize and classify multiple disease lesions within a single image, offering distinct advantages for tomato leaf disease detection under complex and variable field conditions [14,15].

Although extensive research has been conducted on tomato leaf disease detection, several challenges remain to be addressed. For example, Zhao et al. [16] developed a diagnosis method based on an enhanced convolutional neural network that integrates a squeeze-and-excitation (SE) attention mechanism to improve feature extraction and employs data augmentation to enhance model generalization. However, their experiments were limited to controlled laboratory conditions using publicly available datasets, leaving the model’s real-world generalization ability unverified. Saeed et al. [17] adopted a transfer learning strategy using pre-trained Inception V3 and Inception-ResNet V2 models combined with dropout tuning for disease classification under both laboratory and field conditions. Despite achieving promising results, the method was restricted to three categories—Early Blight, Yellow Leaf Curl Virus, and Healthy—thus limiting its applicability. Lu et al. [18] proposed the IMobileTransformer, a lightweight hybrid model featuring a three-branch architecture that effectively integrates MobileNet’s strengths in local feature extraction with Transformer’s capability for global dependency modeling. However, the model may still contain certain redundancies, resulting in increased computational cost and model complexity. Lu et al. [19] introduced a rice yield prediction approach based on a QRBILSTM-MHSA network. This method leverages bidirectional LSTM for time-series feature extraction and employs a multi-head self-attention mechanism to dynamically weight key growth factors, while incorporating quantile regression to predict yield intervals. The model also integrates hyperspectral and multi-source sensor data to improve prediction accuracy and quantify uncertainty. However, its performance is highly sensitive to data quality and can be influenced by measurement device precision and noise from factors such as extreme weather. Wang et al. [20] introduced an attention-based multi-scale feature fusion network incorporating CBAM and a reparameterized BiRepGFPN module, which effectively improved small-lesion detection in complex environments. However, misdetections persisted when visually similar symptoms occurred among small lesions. Ye et al. [21] proposed the Transformer-based Tswin-F network, which integrates bilateral local attention, self-supervised learning, and feature fusion attention mechanisms to achieve efficient multi-scale feature aggregation, substantially improving recognition accuracy. Yet, its complex architecture incurs high computational cost and prolonged training time. Li et al. [22] designed an MFF-CNN model built upon YOLOv5s, embedding coordinate attention and an enhanced spatial pyramid pooling module to improve feature representation and enable real-time detection of corn leaf diseases. Despite these improvements, the model struggles with missed detections for edge or densely clustered targets. Most recently, Wang et al. [23] proposed TomaFDNet, an improved YOLOv8-based framework that incorporates a Multi-Scale Focus Diffusion Network (MSFDNet) and an Efficient Parallel Multi-Scale Convolution (EPMSC) module to strengthen multi-scale feature extraction and small-object detection. Although the model performs well overall, its sensitivity to early-stage, low-contrast disease symptoms remains limited, often leading to false negatives.

In summary, despite the remarkable progress achieved in tomato leaf disease detection, several critical challenges remain. On one hand, the complexity and variability of lesion morphology—together with variations in illumination, shooting angles, and background interference—can significantly degrade detection accuracy. On the other hand, the visual differences among early-stage disease symptoms are often subtle, increasing the difficulty of accurate identification. To address these challenges, this paper proposes an improved detection framework that is robust to complex field environments while maintaining real-time performance. The main contributions of this study are summarized as follows:

• EfficientMSF Module: Enhances multi-scale feature extraction, enabling the model to more effectively identify lesions of varying sizes and shapes, thereby improving detection robustness under diverse environmental conditions.
• C2CU Module: Strengthens global contextual modeling by capturing long-range dependencies among lesions, effectively reducing confusion between diseases with similar visual characteristics.
• CAFMFusion Module: Achieves efficient fusion of local details and global semantic information, enhancing overall feature representation while preserving fine-grained sensitivity, which significantly improves the detection of small lesions and complex background scenes.

## 2. Relevant Work

### 2.1. Evolution and Optimization of Feature Pyramid Networks

The Feature Pyramid Network (FPN) introduced a top–down architecture with lateral connections to fuse features across multiple scales. By progressively upsampling deep semantic features and merging them with shallow high-resolution representations, FPN enables effective multi-scale perception in object detection. However, its unidirectional information flow limits the completeness of feature interaction [24].

To address this limitation, the Path Aggregation Network (PANet) incorporated an additional bottom–up pathway, enhancing the transmission of low-level spatial details to high-level semantic layers and thereby improving small-object detection performance. Nonetheless, this improvement came at the cost of increased computational complexity [25]. Subsequently, NAS-FPN employed neural architecture search to automatically discover optimal feature-fusion connections, achieving substantial accuracy gains but suffering from excessive search and inference costs, which hinder practical deployment [26].

To achieve a better trade-off between accuracy and efficiency, the Bidirectional Feature Pyramid Network (BiFPN) was proposed. BiFPN streamlines the feature-fusion structure by removing redundant connections and introduces bidirectional information flow, enabling semantic and spatial features to interact effectively in both top–down and bottom–up directions. Furthermore, its learnable weighted fusion mechanism adaptively assigns importance to multi-level features, preventing information suppression caused by naive summation. By stacking BiFPN modules iteratively, feature representations can be progressively refined. Owing to its efficiency and scalability, BiFPN has become a key component in modern lightweight detectors such as EfficientDet and represents a significant milestone in the evolution of multi-scale feature fusion from FPN to practical high-performance applications [27].

### 2.2. GhostConv Module

GhostConv [28], the core module of GhostNet, is designed to generate more feature maps through computationally inexpensive operations. This design philosophy aligns with the broader trend in model compression and efficiency research, which aims to reduce redundancy while maintaining competitive performance [29]. The process begins with a primary convolution using a limited number of kernels to extract intrinsic feature maps, serving as the skeleton of the output. This step captures the essential structural information—such as edges and textures—necessary for subsequent feature enrichment. Thereafter, a series of low-cost linear transformations, typically implemented via depthwise convolutions, are applied to these intrinsic features to produce additional ghost features. The use of linear or lightweight operations to expand feature diversity shares a conceptual motivation with the depthwise separable convolutions in MobileNetV2 [30] and the channel shuffle operation in ShuffleNet [31], both of which aim to decouple feature interactions for improved efficiency. By exploiting the inherent redundancy in intermediate feature maps [29,32], GhostConv provides a cost-effective mechanism to increase channel capacity and representational diversity. Finally, the intrinsic and ghost features are concatenated to form the complete output, achieving a balance between efficiency and expressiveness.

### 2.3. YOLO11 Model

YOLO11 represents the latest generation in the YOLO family of object detectors, inheriting the advantages of end-to-end training, real-time inference, and high detection accuracy characteristic of its predecessors. It further optimizes the network architecture and feature-fusion mechanisms to achieve an improved balance between lightweight design and detection robustness [33]. Compared with earlier versions, YOLO11 introduces newly designed modules—such as C3k2 and C2PSA (Parallel Spatial Attention)—to enhance both the backbone and neck structures, thereby improving multi-scale feature representation and small-object detection performance. In addition, to address efficiency and resource constraints, variants of YOLO11 have been developed for different deployment scenarios, as exemplified by YOLO11 Optimization for Efficient Resource Utilization [34], which introduces resource-adaptive model configurations tailored to varying object sizes. These refinements collectively make YOLO11 highly suitable for applications demanding both high accuracy and low latency.

## 3. Method Design

**Figure 1:**
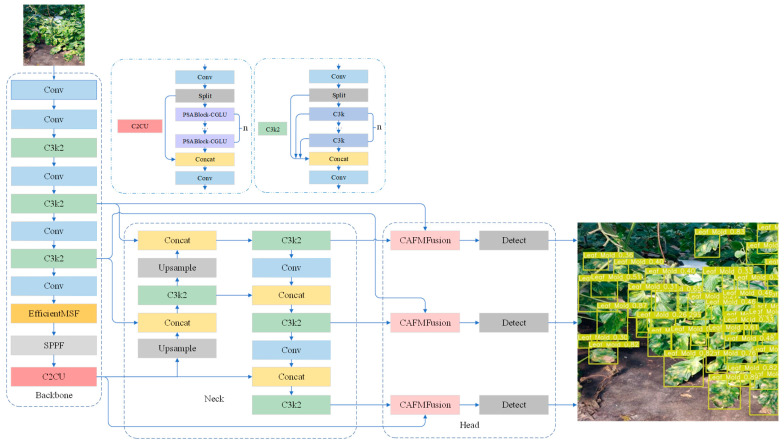
Network structure diagram of improved YOLO11n.

This paper proposes an improved network architecture based on YOLO11n to enhance the robustness and accuracy of tomato leaf disease detection in complex environments. The overall framework is shown in Figure 1, and it primarily introduces three core modules in the feature extraction and feature fusion stages: EfficientMSF, C2CU, and CAFMFusion. First, the EfficientMSF module is embedded in the backbone network to strengthen the model’s ability to represent lesions of different sizes and shapes through a multi-scale feature selection mechanism, thereby improving detection robustness under scale variations and lighting interference. Next, the C2CU module is introduced, which combines position-sensitive attention with global context modeling. This effectively enhances the model’s ability to capture long-range dependencies between features, excelling in distinguishing similar diseases and suppressing complex backgrounds. Finally, the CAFMFusion module is designed, employing a cross-attention fusion strategy between local details and global semantics to efficiently integrate features from different levels. This approach improves overall feature representation while maintaining sensitivity to details, significantly enhancing the recognition accuracy of small lesions and weak-textured targets. This design aims to optimize feature extraction and fusion efficiency while maintaining a balance between detection accuracy and inference speed. The effectiveness of this architecture will be further verified in the experimental results and discussion sections.

### 3.1. EfficientMSF Module

Tomato leaf lesions exhibit considerable variability in size and morphology, making single-scale feature extraction inadequate for accurate detection. To address this limitation, this study designs the EfficientMSF module to enhance the network’s multi-scale feature extraction capability. The module introduces parallel convolutional branches with different receptive fields to capture discriminative information across multiple scales. A lightweight feature weighting mechanism is then employed to adaptively select and fuse features, improving the model’s ability to recognize lesions of diverse scales while maintaining computational efficiency. This design effectively balances detection accuracy and inference speed, ensuring robust performance in complex environmental conditions.

**Figure 2:**
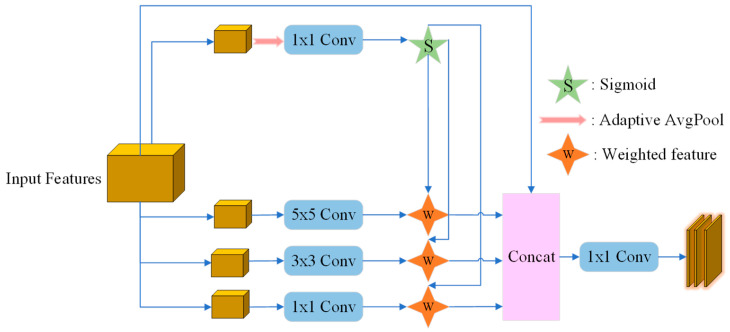
EfficientMSF Module.

The structure of the EfficientMSF module is shown in Figure 2. This module optimizes the feature representation capability through multi-scale feature extraction and adaptive weight adjustment, thereby improving the model’s performance. First, the input feature X is fed into multi-scale convolution branches, where three convolution kernels of different sizes (1 × 1, 3 × 3, 5 × 5) are used to extract features at different scales. The output features of these convolution branches are weighted to emphasize the importance of features at different scales. Meanwhile, the input feature also passes through an adaptive feature selection module, which first performs global average pooling and then outputs three weights through a 1 × 1 convolution layer. These weights, generated by the Sigmoid activation function, are used to adjust the weights of the output features from the multi-scale convolution branches, as shown in Equation (1).

(1) $$F_{weighted}=\{F_{1\times 1}⊙\alpha_{1},F_{3\times 3}⊙\alpha_{2},F_{5\times 5}⊙\alpha_{3}\}$$

In the equation, $F_{k\times k}$ represents the result of the  k×k convolution operation, $\alpha_{i}$ is the corresponding attention weight, and ⊙ denotes element-wise multiplication.

Next, the weighted features from the multi-scale convolution branches are concatenated, and then further integrated through a 1 × 1 convolution layer. Finally, the convolved result is added to the original features to output the fused features, as shown in Equations (2) and (3).

(2) $$F_{concat}=Concat(F_{weighted})$$

(3) $$F_{fused}=Conv_{1\times 1}(F_{concat})+X$$

In the equation, *Concat* represents feature concatenation, and *Conv*_1×1_ denotes the 1 × 1 convolution operation.

### 3.2. C2CU Module

In tomato leaf disease detection, different lesion types often exhibit highly similar visual patterns and indistinct boundaries, making it difficult for models that rely solely on local features to achieve reliable discrimination. To overcome this limitation, the C2CU module is introduced to enhance the network’s global context modeling capability. By integrating a position-sensitive attention (PSA) mechanism with context-guided long-range dependency modeling (CGLU) [35], the module effectively captures remote spatial relationships among lesions while suppressing interference from complex backgrounds and visually similar diseases. This design improves the model’s holistic understanding of lesion distributions and substantially reduces misclassification rates in challenging scenarios involving subtle inter-class variations.

**Figure 3:**
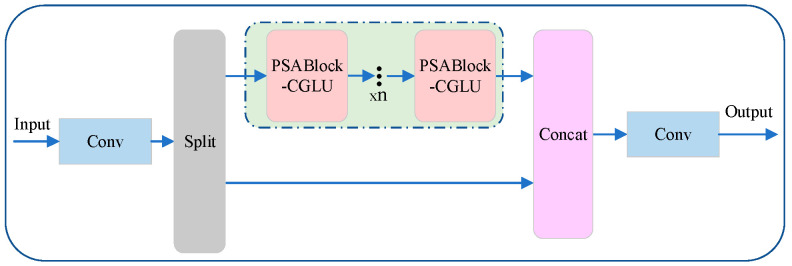
C2CU Module.

Figure 3 illustrates the process of the C2CU module, which is as follows: First, the input feature map X undergoes initial feature extraction through a convolutional layer. Then, the extracted features are split into two parts, $X_{1a}$ and $X_{1b}$, with one part passing through a module group consisting of multiple identical sub-modules (PSABlock-CGLU). After *n* iterations of PSABlock-CGLU processing, these sub-modules capture the dependencies between spatial positions, enhancing the model’s global context awareness. Meanwhile, the other part of the feature directly skips these sub-modules and moves on to subsequent processing. The features $X_{2a}$ processed by the PSABlock-CGLU module and the features $X_{1b}$ that skipped the sub-modules are merged in the Concat module to integrate features extracted from different paths. The merged features $X_{3}$ then go through a convolutional layer for further feature extraction and integration. Finally, the processed data is output as y. As shown in Equations (4)–(8).

(4) $$X_{1}=f_{\mathrm{conv}}(X)$$

(5) $$X_{1a},X_{1b}=f_{\mathrm{split}}(X_{1})$$

(6) $$X_{2a}=f_{\mathrm{psa}}^{n}(X_{1a})$$

(7) $$X_{3}=f_{\mathrm{concat}}(X_{2a},X_{1b})$$

(8) $$y=f_{\mathrm{conv}}(X_{3})$$

In this context, $f_{\mathrm{psa}}$ represents the operation of the PSABlock-CGLU module, X denotes the input feature map, $f_{\mathrm{psa}}^{n}$ indicates the repetition of the $f_{\mathrm{psa}}$ operation $N$ times, $f_{\mathrm{conv}}$ refers to the convolution operation, and $f_{\mathrm{split}}$ represents the splitting operation.

### 3.3. CAFMFusion Module

In tomato leaf disease detection, relying exclusively on either local details or global semantics often results in suboptimal performance. To overcome this limitation, the CAFMFusion module is designed to efficiently integrate local and global feature representations. It employs a cross-attention mechanism to adaptively combine fine-grained local features with high-level semantic information, maintaining sensitivity to small lesions while enhancing overall feature expressiveness. Consequently, the module improves detection accuracy in complex backgrounds and multi-disease coexisting scenarios.

**Figure 4:**
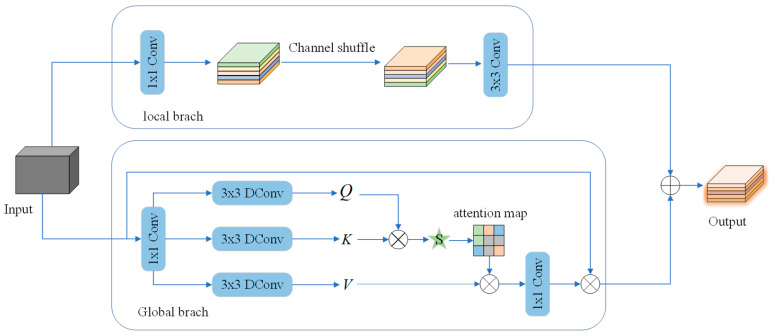
Schematic diagram of the CAFM structure.

As a novel feature-fusion strategy, CAFMFusion enables the network to learn how to selectively extract and integrate valuable contextual information. Through the cross-attention mechanism, the model dynamically aligns global context with locally extracted features, significantly strengthening its capacity to model and represent complex visual patterns. The overall structure of CAFMFusion is illustrated in Figure 4, which adopts a dual-branch design—comprising a Local Branch and a Global Branch—that interact through cross-attention to produce enhanced, semantically enriched features.

In the Local Branch, a 1 × 1 convolution is first applied for channel transformation and dimensional alignment. This is followed by a channel shuffle operation to facilitate inter-group information exchange and increase feature diversity. Finally, three 3 × 3 standard convolutions are employed to aggregate spatial information and extract local structural patterns, as formulated in Equations (9)–(11).

(9) $$X_{local}^{1}=C{\mathrm{onv}}_{1\times 1}(X)$$

(10) $$X_{local}^{2}=Shuffle(X_{local}^{1})$$

(11) $$X_{local}^{out}=C{\mathrm{onv}}_{3\times 3\times 3}(X_{local}^{2})$$

In the equation, $X_{local}^{out}$ represents the output of the local branch, Shuffle denotes the channel shuffle operation, and X refers to the input feature map.

In the global branch, the feature map $X\in {\mathbb{R}}^{H\times W\times C}$ is first processed through convolution 1 × 1 and depthwise convolution 3 × 3 to generate query (Q), key (K), and value (V), resulting in shape $Q,K,V\in R^{H\times W\times C}$. Then, a matrix multiplication is performed to compute the attention, and they are reshaped into $\hat{Q}\in {\mathbb{R}}^{HW\times C}$, $\hat{K}\in {\mathbb{R}}^{C\times HW}$, and $\hat{V}\in R^{HW\times C}$. The attention matrix $\hat{Q}$ is then computed through the interaction between $\hat{K}$ and $A_{\mathrm{attention}}$, and the final output $F_{att}$ is generated through a 1 × 1 convolution. Finally, the global branch output is fused with the local branch output to produce $F_{out}$, as shown in Equations (12)–(14).

(12) $$F_{\mathrm{att}}={\mathrm{Conv}}_{1\times 1}(A_{\mathrm{attention}}(\hat{Q},\hat{K},\hat{V}))+X$$

(13) $$A_{\mathrm{attention}}(\hat{Q},\hat{K},\hat{V})=\hat{V}\mathrm{Softmax}(\hat{Q}\hat{K}/\alpha )$$

(14) $$F_{out}=F_{att}+X_{local}^{out}$$

In the equation, α is a learnable scaling parameter used to control the magnitude of the multiplication with QK, preventing the Softmax from becoming too smooth or oversaturated. $F_{\mathrm{out}}$ is the final output of the CAFM module.

**Figure 5:**
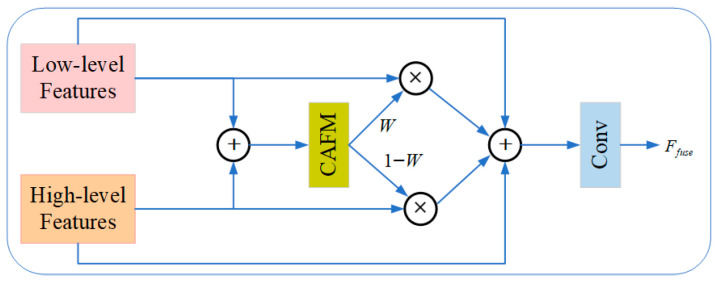
CAFMFusion module.

Figure 5 shows the entire CAFM Fusion [36]. The CAFM Fusion mechanism is a technique for fusing low-level and high-level features. First, it receives two types of feature inputs: low-level features and high-level features. These features are passed into the CAFM module, which is responsible for aggregating contextual information and outputs two weighted features, labeled as w and 1−w, respectively. Next, these two weighted features are multiplied by the original low-level and high-level features to adjust the weights of the features. Then, the weighted features are added together to merge the information. Finally, the merged features undergo further processing through a convolutional layer with 1×1 to obtain the final fused feature $F_{\mathrm{fuse}}$. This process aims to enhance the model’s performance by effectively combining features from different levels, as shown in Equation (15).

(15) $$F_{\mathrm{fuse}}={\mathrm{Conv}}_{1\times 1}(F_{\mathrm{low}}⊕(F_{\mathrm{low}}\times w)⊕(F_{\mathrm{high}}\times (1-w))⊕F_{\mathrm{high}})$$

In the equation, $F_{\mathrm{low}}$ represents the low-level features. $F_{\mathrm{high}}$ represents the high-level features. w is the weight output by the CAFM module. ⊕ represents the additive fusion of features. × represents the element-wise multiplication of features.

## 4. Experiment

### 4.1. Dataset

The dataset comprises 2212 high-resolution images of tomato leaves collected in the Sanya region of Hainan, China, encompassing nine representative conditions: (a) Early Blight, (b) Healthy, (c) Late Blight, (d) Leaf Miner, (e) Leaf Mold, (f) Mosaic Virus, (g) Septoria, (h) Spider Mites, and (i) Yellow Leaf Curl Virus, along with other common disease types. The dataset is divided into 1769 training images and 443 test images, providing a diverse and well-balanced foundation for model training and evaluation.

Images were captured across different growth stages and seasons between 8:00 AM and 5:00 PM, incorporating natural variations in illumination and environmental context. To ensure consistency, all samples were collected under stable weather conditions, and low-quality images—such as those that were blurry, over- or under-exposed, or lacked visible targets—were excluded through rigorous quality control. Background interference was minimized by focusing on single-leaf compositions and leveraging natural depth-of-field effects. All photographs were taken using a Huawei Pura 70 smartphone (50 MP main camera), ensuring high pixel fidelity and detailed imaging capable of capturing subtle disease characteristics such as lesion morphology, color gradation, and texture variation. During the data preprocessing stage, the collected images were carefully filtered to reduce data interference and improve data quality. Through strict quality assessment, low-quality images (such as blurred images, overexposed or underexposed images) and images lacking the target were excluded, ensuring the integrity and reliability of the dataset, and further enhancing its usability. Image annotations were produced using the LabelImg tool following standardized labeling protocols. Each sample was precisely tagged with its corresponding disease category, providing accurate reference data for supervised learning and diagnostic validation. The careful data curation and preprocessing steps ensured the dataset’s integrity, reliability, and practical usability.

**Figure 6:**
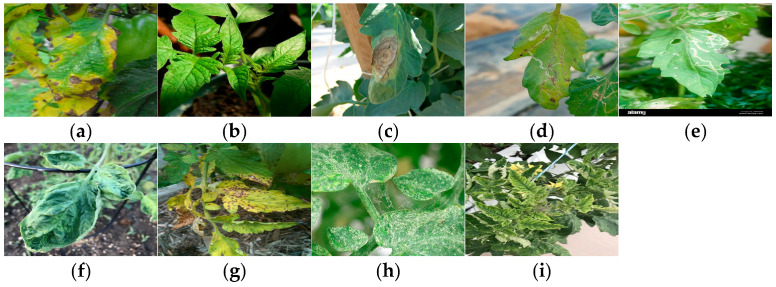
Sample images of tomato leaves of different categories.(**a**) Early Blight, (**b**) Healthy, (**c**) Late Blight, (**d**) Leaf Miner, (**e**) Leaf Mold, (**f**) Mosaic Virus, (**g**) Septoria, (**h**) Spider Mites, and (**i**) Yellow Leaf Curl Virus.

To facilitate reader identification and correlate with the sample images in Figure 6, brief descriptions of the nine tomato leaf conditions are provided below, based on standard phytopathological references: (a) Early Blight: Caused by the fungus Alternaria solani, symptoms include dark brown concentric rings on older leaves, often surrounded by a yellow halo. (b) Healthy: Leaves exhibit no signs of disease or stress, characterized by uniform green coloration and intact structure. (c) Late Blight: Resulting from infection by Phytophthora infestans, it is characterized by water-soaked, greasy-appearing lesions that may turn brown and necrotic. (d) Leaf Miner: Damage caused by larvae of insects such as Liriomyza spp., creating distinctive winding, white trails or “mines” within the leaf tissue. (e) Leaf Mold: Caused by Fulvia fulva, symptoms include pale green or yellow patches on the upper leaf surface and a distinctive purple-brown mold growth on the underside. (f) Mosaic Virus: Caused by pathogens like Tomato mosaic virus (ToMV), leading to characteristic light and dark green mottling (mosaic patterns), leaf distortion, and stunted growth. (g) Septoria: Caused by the fungus Septoria lycopersici, identified by numerous small circular spots with dark margins and lighter centers, typically found on lower leaves. (h) Spider Mites: Infestation by mites such as Tetranychus spp. causes stippling (tiny yellow dots), yellowing, and sometimes fine webbing on the leaf undersides. (i) Yellow Leaf Curl Virus: A geminivirus (TYLCV) that causes upward curling of leaves, yellowing (chlorosis), and significant stunting of the plant.

Data collection covered the full tomato growth cycle—from seedling to maturity—capturing disease manifestations across developmental stages. Owing to its tropical monsoon climate characterized by high humidity, abundant rainfall, and year-round warm temperatures, the Sanya region presents favorable conditions for the proliferation of fungal and viral pathogens such as Alternaria solani (Early Blight), Phytophthora infestans (Late Blight), and Tomato Yellow Leaf Curl Virus. These climatic factors accelerate disease progression and frequently lead to overlapping infections, making Sanya a representative site for studying complex multi-disease interactions in tomato crops. Representative sample images of the nine categories are shown in Figure 6, and the statistics of the plant disease classification images are presented in Table 1.

### 4.2. Experimental Platform and Hyperparameter Setting

The experiments were conducted using PyTorch 1.10.0 as the deep learning framework under a Python 3.8 environment on Ubuntu 20.04. GPU acceleration was enabled through CUDA 11.3. The training hardware consisted of a single NVIDIA RTX 4090 GPU (24 GB), an AMD EPYC 7T83 64-core processor (22 vCPUs), and 90 GB of system memory, providing sufficient computational resources for model training and evaluation.

For the training configuration, input images were resized to 640 × 640, with a batch size of 64 and 200 training epochs. The optimizer parameters were set as follows: an initial learning rate of 0.01, momentum of 0.937, and a weight decay coefficient of 0.0005. In addition, eight data-loading workers were employed to enhance training efficiency. This configuration ensured stable convergence and efficient utilization of hardware resources throughout the training process.

### 4.3. Evaluation Indicators

The experiments in this paper use F1 score, Precision (P), Recall (R), Average Precision (AP), and mean Average Precision (mAP) as evaluation metrics [37], and also refer to the number of parameters (Parameters). The calculation expressions for these metrics are shown in Equations (16)–(20).

(16) $$\mathrm{Precision}=\frac{Tp}{Tp+Fp},$$

(17) $$\mathrm{Recall}=\frac{Tp}{Tp+FN},$$

(18) $$\mathrm{AP}=\int_{0}^{1}P(R)dR,$$

(19) $$\mathrm{mAP}=\frac{1}{n}\sum_{i=0}^{n}AP(i),$$

(20) $$F1=\frac{2\times \mathrm{Precision}\times \mathrm{Recall}}{\mathrm{Precision}+\mathrm{Recall}},$$

In these equations, Tp represents the number of correctly detected objects; Fp represents the number of incorrectly detected objects; FN represents the number of missed objects; n denotes the number of categories; and AP(i) represents the average precision for the i-th object class.

## 5. Experimental Analysis

### 5.1. Algorithm Comparison Results

To comprehensively evaluate the effectiveness of the proposed method, a series of systematic experiments were conducted using representative benchmark algorithms under unified data preprocessing and training/inference settings. The comparative models include the Transformer-based RT-DETR-r18 [38], Faster R-CNN [39], SSD [40] and several state-of-the-art lightweight variants from the YOLO family—YOLOv8n [41], YOLOv10n [42], YOLO11n, and YOLOv12n [43]. To achieve a balanced assessment of detection accuracy and computational efficiency, the evaluation metrics include Recall, mAP@0.5, mAP@0.5–0.95, and frames per second (FPS). The overall comparison results are summarized in Table 2.

Table 2 summarizes the performance comparison of different algorithms on the tomato leaf disease detection task. Among the classical detectors, Faster R-CNN achieves a competitive mAP@0.5 of 72.13%, demonstrating strong localization and classification capabilities, though its inference speed is not reported and is generally known to be slower than single-stage detectors. SSD attains a Recall of 70.79% and mAP@0.5 of 73.52%. The proposed method demonstrates a notable improvement in detection accuracy over existing mainstream models. Specifically, it achieves 71.0% Recall, 76.5% mAP@0.5, and 60.5% mAP@0.5–0.95, representing the highest values among all compared methods. Relative to YOLO11n, the proposed approach yields gains of +3.4%, +1.3%, and +2.0% in these three metrics, respectively; compared with RT-DETR-r18, the improvements reach +3.3%, +5.3%, and +3.7%. These results indicate a substantial enhancement in both target recall capability and overall detection precision. In terms of inference speed, the proposed model achieves 400 FPS, which is slightly lower than the 454.5 FPS achieved by YOLO11n and YOLOv12n, but markedly higher than the 133.3 FPS of RT-DETR-r18 and the 303 FPS of YOLOv8n and YOLOv10n. Overall, the proposed architecture achieves a superior balance between accuracy and efficiency, delivering significant improvements in detection performance while maintaining real-time inference capability. These findings highlight its strong potential for practical deployment in intelligent agricultural monitoring systems.

Table 3 presents a detailed comparison of category-wise detection accuracy between the proposed algorithm and several mainstream object detection models, including RT-DETR-r18, YOLOv8n, YOLOv10n, YOLO11n, and YOLOv12n. The results show that the proposed method consistently achieves the highest detection accuracy across all seven plant disease categories. Specifically, the model attains 78.1% accuracy for Late Blight, exceeding YOLO11n’s 77.5%; and 84.6% for Leaf Mold, representing a +3.4% improvement over YOLO11n (81.2%). For Mosaic Virus, the accuracy reaches 89.3%, outperforming YOLO11n (87.9%) by +1.4%. In Septoria, the proposed model achieves 56.0%, slightly higher than 55.5% from YOLO11n. Similarly, for Leaf Miner and Spider Mites, accuracies of 94.5% and 88.8% are obtained, surpassing YOLO11n’s 93.6% and 87.4%, respectively. Finally, in the Healthy category, the model achieves 79.5%, outperforming YOLO11n (76.5%) with a relative improvement of +3.0%. These results confirm that the proposed method maintains superior detection capability across diverse disease categories, particularly for Leaf Mold and Mosaic Virus, which are characterized by subtle lesion textures and inter-class visual similarity. This demonstrates the model’s enhanced discriminative power and robustness in handling fine-grained agricultural disease features.

Table 4 compares the category-wise Recall of the proposed method with several representative object detection algorithms. The results show that the proposed model achieves the highest Recall in the Healthy (83.9%), Leaf Miner (87.1%), and Septoria (50.8%) categories, exhibiting notable gains over other methods. In particular, Recall for the Healthy category increases by +7.3% compared with YOLOv12n, while in the Leaf Miner category the proposed model achieves superior performance relative to both RT-DETR-r18 and the YOLO series. For Septoria, the Recall of 50.8% surpasses RT-DETR-r18 by +3.1% and YOLO11n by +5.9%, significantly improving detection sensitivity for this disease. Although the Recall in the Leaf Mold category (79.2%) is slightly lower than that of YOLOv12n (81.1%), it still outperforms all other compared methods. Overall, the proposed approach demonstrates leading Recall performance across most categories, confirming its enhanced capability in capturing true positives and maintaining high detection accuracy under complex visual conditions.

Table 5 presents the cross-dataset evaluation results on the VisDrone and PASCAL VOC datasets, showing that the proposed model achieves consistent and comprehensive performance improvements over the baseline YOLO series. On the VisDrone dataset, which is characterized by small, densely distributed, and low-contrast objects, our method obtains a Recall of 34.4%, mAP@0.5 of 34.2%, and mAP@0.5–0.95 of 19.8%, surpassing YOLOv8n to YOLOv12n by 1.0–4.5 percentage points in overall detection accuracy. On the PASCAL VOC dataset, which contains objects of moderate size and a balanced category distribution, the proposed model achieves a Recall of 45.1%, mAP@0.5 of 48.2%, and mAP@0.5–0.95 of 29.3%, corresponding to gains of +0.8%, +1.5%, and +1.4% over the best-performing baseline YOLO11n, respectively. These consistent improvements across two benchmark datasets with distinct characteristics validate the strong generalization capability, robustness, and applicability of the proposed architecture in diverse object detection scenarios.

Table 6 presents a comparative analysis of computational efficiency among different algorithms, including GFLOPS, GPU memory usage, and inference speed (FPS). As shown, RT-DETR-r18 exhibits the highest computational complexity, requiring 13.0 GB of GPU memory and achieving only 133.3 FPS, indicating a substantial trade-off between accuracy and speed. In contrast, the lightweight YOLO variants (YOLOv8n and YOLOv10n) effectively reduce computational overhead to approximately 8 GFLOPS, reaching 303 FPS under identical hardware conditions. The proposed model demonstrates superior efficiency, requiring only 7.9 GFLOPS and 9.0 GB of GPU memory while achieving the highest inference speed of 400 FPS. These results confirm that the introduced architectural optimizations significantly enhance computational efficiency without compromising detection accuracy, thereby ensuring real-time performance and suitability for deployment in resource-constrained environments.

### 5.2. Visualization of Results

To provide a more comprehensive interpretation of the overall and category-specific results summarized in Table 1, Table 2 and Table 3, we further analyze the confusion matrix normalized of each model. Unlike single-value metrics such as mAP or Recall, the confusion matrix simultaneously illustrates both the true positive rate and the misclassification distribution across categories, thereby visualizing how different models handle inter-class confusion and background interference. This representation offers a clearer understanding of each model’s category discrimination capability and robustness, enabling more accurate and intuitive comparisons among competing algorithms.

**Figure 7:**
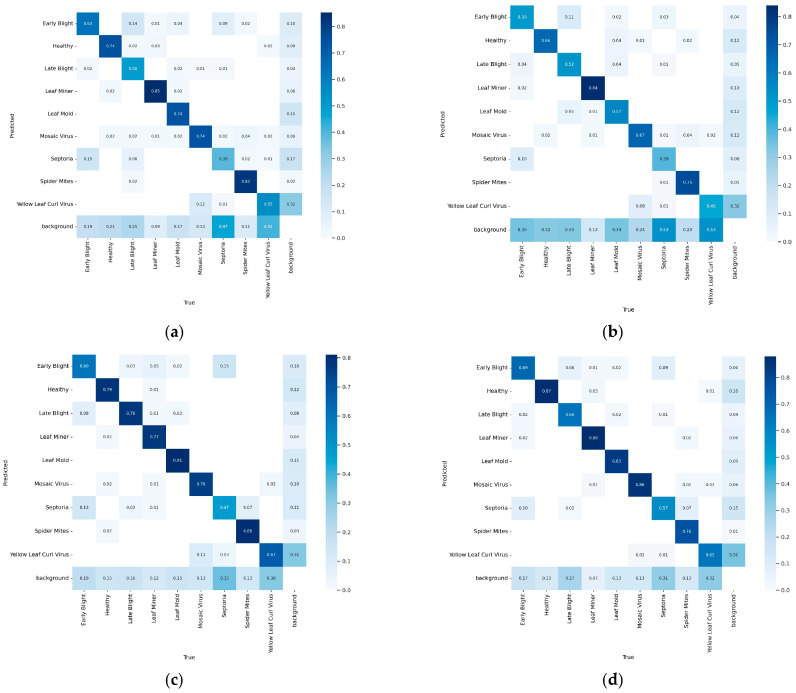
Confusion matrix normalized: (**a**) YOLOv8n; (**b**) YOLOv10n; (**c**) YOLOv12n; (**d**) Ours.

From the overall distribution of the confusion matrices in Figure 7, the proposed model exhibits a notably darker diagonal and lighter off-diagonal regions, indicating higher overall classification accuracy, lower inter-class confusion, and stronger category discrimination compared with YOLOv8n, YOLOv10n, and YOLOv12n. For the Healthy and Leaf Miner categories, the proposed method achieves recognition rates of approximately 0.87 and 0.88, respectively, outperforming all compared YOLO variants and demonstrating stable detection performance for common disease types. In the Leaf Mold and Mosaic Virus categories, accuracies of 0.83 and 0.86 are obtained, surpassing YOLOv12n’s 0.81 and 0.76, and substantially reducing misclassification with visually similar lesions. The improvement is particularly pronounced in the more challenging Septoria category, where the proposed model raises accuracy from 0.47 (YOLOv12n) to 0.57, and achieves an even larger gain over YOLOv8n (0.39) and YOLOv10n (0.39), highlighting its superior robustness in small-sample and easily confused classes. For Yellow Leaf Curl Virus, the proposed model attains an accuracy of 0.65, comparable to YOLOv12n’s 0.67, yet clearly outperforming YOLOv8n (0.55) and YOLOv10n (0.45), effectively reducing background misclassification. In contrast, YOLOv10n exhibits frequent false detections in background regions, while YOLOv8n and YOLOv12n still show considerable confusion between Septoria and Leaf Mold. Overall, the proposed model achieves higher intra-class hit rates and markedly lower inter-class confusion across most categories. Its superior performance in complex, visually similar, and small-sample classes demonstrates enhanced accuracy, stronger confusion suppression, and improved robustness in practical object detection scenarios.

**Figure 8:**
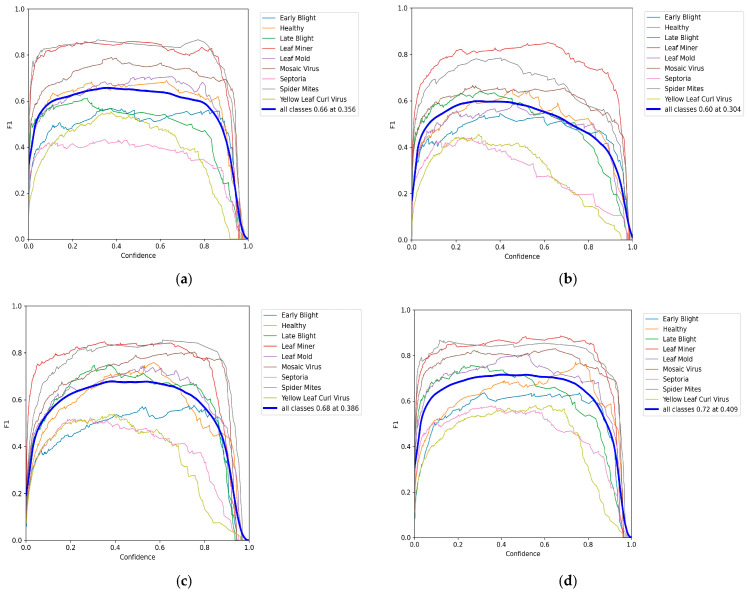
F1-Confidence curves: (**a**) YOLOv8n; (**b**) YOLOv10n; (**c**) YOLOv12n; (**d**) Ours.

Figure 8 illustrates the F1–Confidence curves, which reflect the trade-off between precision and recall across varying confidence thresholds. The comparative results show that the highest average F1-scores achieved by YOLOv8n, YOLOv10n, and YOLOv12n are 0.66, 0.60, and 0.68, respectively, whereas the proposed model attains the best performance with an average F1 of 0.72. From the overall curve trend, the proposed method maintains not only a consistently higher F1 trajectory but also exhibits reduced fluctuation, indicating superior stability across thresholds. Category-wise analysis further reveals that the proposed model consistently outperforms the YOLO series in both common disease categories—such as Leaf Miner and Mosaic Virus—and more challenging ones, including Septoria and Yellow Leaf Curl Virus. This highlights its enhanced robustness in small-sample and visually confounding categories. Overall, the smoother and higher F1–Confidence curve of the proposed model demonstrates an improved balance between precision and recall, further validating its overall detection superiority and reliability in complex agricultural scenarios.

**Figure 9:**
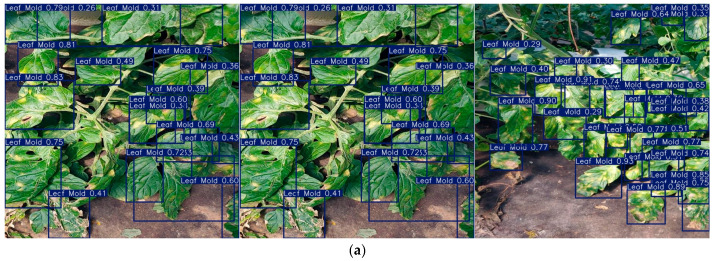

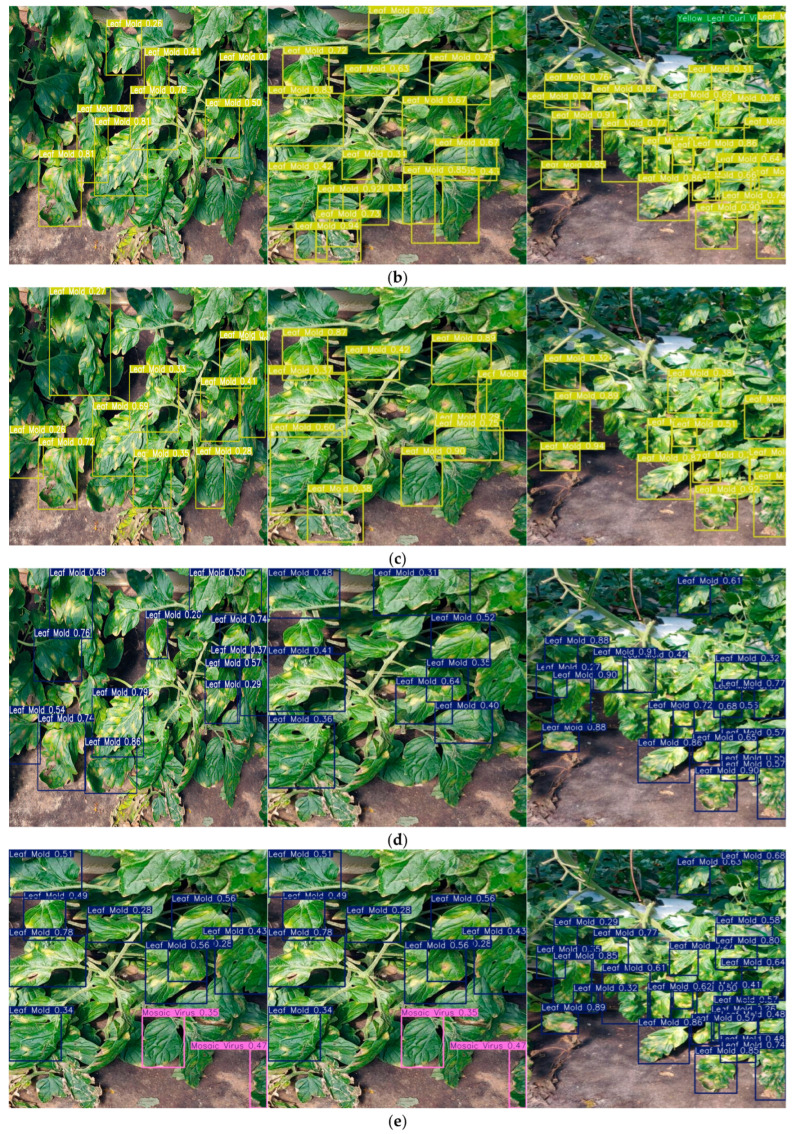

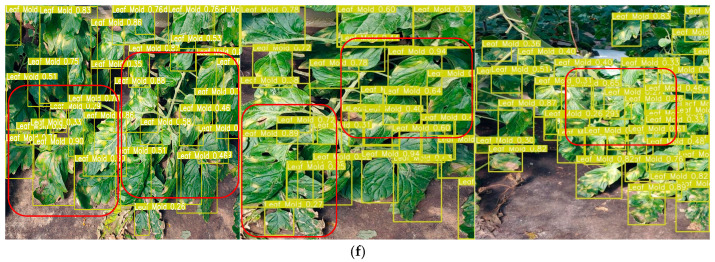
Detection performance of different algorithms on the dataset: (**a**) RT-DETR-r18; (**b**) YOLOv8n; (**c**) YOLOv10n; (**d**) YOLO11n; (**e**) YOLOv12n; (**f**) Ours.

Figure 9 presents a qualitative comparison of different detectors for greenhouse tomato disease recognition. To evaluate their detection behavior under identical conditions, the same high-density leaf scene was divided into three subregions—left, middle, and right—and the prediction results of RT-DETR-r18, YOLOv8n, YOLOv10n, YOLO11n, YOLOv12n, and the proposed model were overlaid. The comparison focuses on recall, precision, duplicate suppression, category distinction, confidence distribution, and bounding-box consistency under challenging conditions such as strong reflections, shadows, overlapping leaves, and early-stage lesions. Special attention is given to false detections induced by leaf veins and specular highlights, the coverage of small marginal lesions under backlighting, and category stability in multi-disease coexistence scenarios. (a) RT-DETR-r18: Produces densely packed deep-blue bounding boxes, often exhibiting “boxes within boxes” and redundant detections of the same lesion. The high sensitivity to strong reflections and vein contrast yields high recall but frequent over-detection and numerous low-confidence predictions. (b) YOLOv8n: Generates cleaner yellow boxes with fewer overlaps and improved focus on leaf edges and large lesions. However, small marginal or backlit lesions are occasionally missed, and recognition of minority classes such as Yellow Leaf Curl Virus remains limited. (c) YOLOv10n: Achieves a more balanced trade-off between detection coverage and visual neatness, with box sizes closely matching lesion areas. Nevertheless, moderate confidence dispersion and noticeable missed detections persist. (d) YOLO11n: Further suppresses noise, yielding fewer low-confidence boxes and stable detection of medium-to-large lesions across all regions. False detections from specular highlights are rare, making it well suited for accurate lesion localization and counting. (e) YOLOv12n: Maintains a similar detection clarity to YOLO11n but introduces pink boxes for Mosaic Virus, occasionally leading to false positives—particularly misclassifying Leaf Mold as Mosaic Virus. (f) Proposed (Ours): Produces clean yellow boxes comparable to YOLO11n while achieving slightly higher recall than YOLOv10n/11n. It provides superior coverage of small lesions along edges and under backlighting, effectively avoiding duplicate detections and false positives from vein structures. For large and medium lesions, the predicted boxes are compact and geometrically aligned with lesion contours. Confidence values concentrate between 0.55 and 0.88, with minimal low-confidence detections. In scenes with coexisting diseases, the model maintains stable classification for Leaf Mold and avoids confusing chlorotic patterns with Mosaic Virus. Overall, the proposed model accurately identifies lesions across scales, enhances small-lesion detection, and preserves high precision and category stability in complex visual environments. These results highlight its superior robustness and practicality for real-world agricultural disease monitoring.

**Figure 10:**
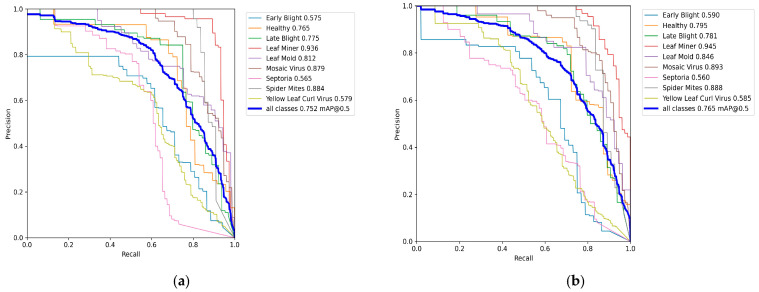
Precision-Recall (PR) curves: (**a**) YOLO11n algorithm; (**b**) Ours.

To further assess the performance gains introduced by the proposed model, a detailed comparison with YOLO11n was conducted across multiple disease categories. As shown in Figure 10, the mAP@0.5 values and Precision–Recall (PR) curves demonstrate clear performance improvements in most categories, particularly within the mid-to-high recall range. Overall, the proposed model achieves an increase in mAP@0.5 from 0.752 to 0.765, representing a +1.3% improvement. n category-level comparisons, Leaf Mold exhibits the most significant gain, improving from 0.812 to 0.846 (+3.4%), followed by Healthy, which rises from 0.765 to 0.795 (+3.0%). Moderate improvements are also observed in Leaf Miner, Mosaic Virus, Late Blight, Spider Mites, and Yellow Leaf Curl Virus, while a slight decline of –0.5% occurs in Septoria (from 0.565 to 0.560). s shown in the PR curves, the proposed model maintains trajectories that lie consistently closer to the upper-right corner in the 40–80% recall range, indicating that false positives are effectively suppressed while preserving recall. These results confirm that the proposed model achieves a better balance between precision and recall and exhibits greater robustness in distinguishing visually similar categories, making it more suitable for reliable real-world deployment.

### 5.3. Ablation Experiment

To further validate the effectiveness and individual contribution of each proposed module, a series of ablation experiments were conducted. The components were incrementally introduced to the baseline model to evaluate their impact on Recall, mAP@0.5, and mAP@0.5–0.95. Table 7 summarizes the detailed results of each experimental configuration, enabling a clear comparison of performance variations across different setups.

Table 7 presents the results of the ablation experiments conducted under identical configurations. The baseline model (YOLO11n) achieves a recall of 67.6%, mAP@0.5 of 75.2%, and mAP@0.5–0.95 of 58.5%. After adding the EfficientMSF module, the recall improves to 69.2%, mAP@0.5 increases to 76.0%, and mAP@0.5–0.95 rises to 59.3%. Combining with the CAFMFusion module further improves these metrics to 70.0%, 76.2%, and 59.5%, respectively. Incorporating the C2CU module results in 70.6%, 76.4%, and 59.8%. Finally, when all three modules (EfficientMSF, C2CU, and CAFMFusion) are used together, recall reaches 71.0%, mAP@0.5 increases to 76.5%, and mAP@0.5–0.95 improves to 60.5%. These results demonstrate that each module contributes positively to the overall detection performance.

In addition, to verify the reliability of the ablation results, we performed multiple repeated experiments under identical configurations. All random seeds, data loading orders, and GPU computation modes were strictly fixed to ensure deterministic training and evaluation. As a result, each repeated run produced identical numerical outcomes without any fluctuation across Recall, mAP@0.5, and mAP@0.5–0.95. This complete reproducibility indicates that the improvements observed in Table 6 are stable and reliable, not caused by random variations. Therefore, confidence intervals or standard deviations are not applicable in this case, since all experimental outputs remained consistent across repeated trials. These results collectively confirm that the introduction of the EfficientMSF, C2CU, and CAFMFusion modules effectively enhances the model’s robustness, stability, and accuracy for tomato leaf disease detection in complex environments.

## 6. Discussion

The experimental results demonstrate that the proposed model achieves significant improvements in detection accuracy and robustness over existing lightweight detectors such as YOLOv8n, YOLOv10n, and YOLO11n. Through the integration of the EfficientMSF, C2CU, and CAFMFusion modules, the network effectively enhances multi-scale feature extraction and global–local information fusion, thereby improving discrimination among visually similar disease categories and achieving reliable detection under complex environmental conditions. The visualization results further confirm that the model substantially reduces misclassification and exhibits superior capability in identifying small and subtle lesions that conventional models often fail to detect.

Future research could extend this work by integrating hyperspectral remote sensing for earlier and more precise disease diagnosis. Since biochemical and structural variations—such as changes in chlorophyll concentration and water content—typically precede visible symptoms, hyperspectral imaging can capture these early physiological signals across narrow spectral bands. Incorporating such spectral information into the improved YOLO11n framework would enable multi-modal fusion of spectral and spatial cues, thereby enhancing early-stage detection sensitivity and environmental robustness.

To further analyze the model’s limitations, a detailed error assessment was conducted on minority categories such as Septoria and Yellow Leaf Curl Virus. These categories contain fewer samples and share high visual similarity in color and morphology with diseases such as Leaf Mold and Mosaic Virus. As illustrated in Figure 7, most misclassifications occur among fungal diseases with overlapping lesion textures or in weak-feature regions where lesions are distributed along leaf margins. Future work could address these challenges by: (1) implementing targeted data augmentation to improve minority-class diversity; (2) adopting dynamic loss re-weighting strategies such as focal or class-balanced loss to emphasize underrepresented categories; and (3) exploring few-shot or generative data expansion using diffusion- or GAN-based approaches to reduce manual annotation demands.

## 7. Conclusions

This paper proposes an improved YOLO11n-based detection method to address the challenges of low detection accuracy, category confusion, and high real-time requirements in tomato leaf disease detection under complex environments. By incorporating three key modules—EfficientMSF, C2CU, and CAFMFusion—into the backbone and feature fusion stages, the model enhances multi-scale feature extraction, global context modeling, and the integration of local and global features. Experiments conducted on a self-built 9-class tomato leaf disease dataset demonstrate that the proposed method achieves a Recall of 71.0%, mAP@0.5 of 76.5%, and mAP@0.5–0.95 of 60.5%, showing significant improvements over the baseline YOLO11n model. Moreover, the model maintains an inference speed of 400 FPS, achieving an effective balance between accuracy and efficiency. The integration of the three modules not only improves feature extraction and fusion efficiency but also ensures better coordination between detection performance and real-time capability, providing a practical solution for real-time tomato leaf disease detection.

Ablation experiments further validate the effectiveness of the improved modules, indicating that the proposed module design plays an active role in enhancing detection performance. Through comprehensive comparison experiments and visualization results, the method demonstrates stronger robustness and generalization ability under complex lighting, background interference, and similar disease symptoms. The research provides reliable technical support for intelligent monitoring of tomato diseases and offers references for optimizing and designing detection methods for other crop diseases. Future work can further explore areas such as lightweight deployment, cross-crop generalization, and multi-modal fusion to promote the widespread application of agricultural disease detection technology in practical production.

## Figures and Tables

**Table 1:** Plant Disease Classification Image Statistics.

Classes	Image Count	Target Count
Early Blight	387	782
Healthy	197	736
Late Blight	244	473
Leaf Miner	383	836
Leaf Mold	224	791
Mosaic Virus	430	654
Septoria	140	598
Spider Mites	121	498
Yellow Leaf Curl Virus	86	1213

**Table 2:** Comparison Results of Experiments with Different Algorithms.

Algorithms	Recall/%	mAP@0.5/%	mAP@0.5–0.95/%	FPS
Faster R-CNN		72.13		
SSD	70.79	73.52		
RT-DETR-r18	67.7	71.2	56.8	133.3
YOLOv8n	65.0	68.7	52.7	303.0
YOLOv10n	55.9	61.5	46.4	303.0
YOLO11n	67.6	75.2	58.5	454.5
YOLOv12n	68.5	72.5	55.1	454.5
Ours	71.0	76.5	60.5	400.0

**Table 3:** Comparison of various algorithms on Average Precision (AP/%).

	Algorithms	RT-DETR-r18	YOLOv8n	YOLOv10n	YOLO11n	YOLOv12n	Ours
Classes							
Healthy		60.3	73.2	66.3	76.5	75.7	79.5
Late Blight		67.2	60.1	62.7	77.5	77.3	78.1
Leaf Miner		92.0	92.3	89.2	93.6	90.4	94.5
Leaf Mold		66.4	72.6	61.1	81.2	80.0	84.6
Mosaic Virus		88.0	79.0	69.8	87.9	83.0	89.3
Septoria		50.1	39.3	36.0	55.5	49.8	56.0
Spider Mites		83.4	87.1	77.3	87.4	85.3	88.8

**Table 4:** Comparison Results of multiple Algorithms in terms of Recall (R/%).

	Algorithms	RT-DETR-r18	YOLOv8n	YOLOv10n	YOLO11n	YOLOv12n	Ours
Classes							
Healthy		58.2	68.5	63.8	72.3	76.6	83.9
Leaf Miner		85.7	85.7	84.0	82.3	78.7	87.1
Leaf Mold		64.1	75.5	52.8	75.5	81.1	79.2
Septoria		47.7	37.1	31.5	44.9	41.6	50.8

**Table 5:** Results of experiments conducted on the VisDrone and PASCAL VOC datasets.

Datasets	Algorithms	Recall	mAP@0.5	mAP@0.5–0.95
VisDrone	YOLOv8n	33.4	32.7	18.9
	YOLOv10n	29.9	29.7	16.5
	YOLO11n	33.4	32.4	18.7
	YOLOv12n	30.9	30.3	17.4
	Ours	34.4	34.2	19.8
PASCAL VOC	YOLOv8n	33.6	33.7	19.0
	YOLOv10n	27.9	24.9	14.2
	YOLO11n	44.3	46.7	27.9
	YOLOv12n	43.4	44.0	26.4
	Ours	45.1	48.2	29.3

**Table 6:** Computational Efficiency Comparison of Different Object Detection Models.

Algorithms	GFLOPS	GPU Mem (GB)	FPS
RT-DETR-r18	57.0	13.0	133.3
YOLOv8n	8.1	10.1	303.0
YOLOv10n	8.2	11.5	303.0
Ours	7.9	9.0	400.0

**Table 7:** Results of Ablation Experiment.

Number	Experiments	Recall/%	mAP@0.5/%	mAP@0.5–0.95/%
1	YOLO11n	67.6	75.2	58.5
2	YOLO11n + EfficientMSF	69.2	76.0	59.3
3	YOLO11n + CAFMFusion	70.0	76.2	59.5
4	YOLO11n + C2CU	70.6	76.4	59.8
5	YOLO11n + EfficientMSF + C2CU + CAFMFusion	71.0	76.5	60.5

## Data Availability

The dataset generated during the current study is available from the corresponding author upon reasonable request.

## Abbreviations

The following abbreviations are used in this manuscript:

YOLO11	You Only Look Once 11
CBAM	Convolutional Block Attention Module
CAFM	Convolution and Attention Fusion Module
EfficientMSF	Efficient Multi-Scale Feature
FPN	Feature Pyramid Network
PANet	Path Aggregation Network
BiFPN	Bidirectional Feature Pyramid Network
CNNs	Convolutional Neural Networks
